# Ten Years of Gabor-Domain Optical Coherence Microscopy

**DOI:** 10.3390/app9122565

**Published:** 2019-06-24

**Authors:** Cristina Canavesi, Jannick P. Rolland

**Affiliations:** 1 LighTopTech Corp., 150 Lucius Gordon Drive, Suite 201, West Henrietta, NY 14586-9687, USA;; 2 The Institute of Optics, University of Rochester, Rochester, NY 14627, USA

**Keywords:** optical coherence tomography, noninvasive imaging, Gabor-domain optical coherence microscopy

## Abstract

Gabor-domain optical coherence microscopy (GDOCM) is a high-definition imaging technique leveraging principles of low-coherence interferometry, liquid lens technology, high-speed imaging, and precision scanning. GDOCM achieves isotropic 2 μm resolution in 3D, effectively breaking the cellular resolution limit of optical coherence tomography (OCT). In the ten years since its introduction, GDOCM has been used for cellular imaging in 3D in a number of clinical applications, including dermatology, oncology and ophthalmology, as well as to characterize materials in industrial applications. Future developments will enhance the structural imaging capability of GDOCM by adding functional modalities, such as fluorescence and elastography, by estimating thicknesses on the nano-scale, and by incorporating machine learning techniques.

## 1. Introduction

Histopathology, the gold standard for diagnosis at the cellular level, suffers from morbidity, cost, and time associated with a biopsy; overcoming these limitations with optical biopsy is the holy grail. The capability to noninvasively image the cellular structures in real-time will revolutionize medicine. The requirements for optical biopsy include cellular resolution (<5 μm) in 3D, 1 mm^2^ field of view, and depth of imaging of at least 1 mm in tissue. Real-time operation is desired.

## 2. Strategies for Cellular-Resolution Imaging

**Figure 1.**
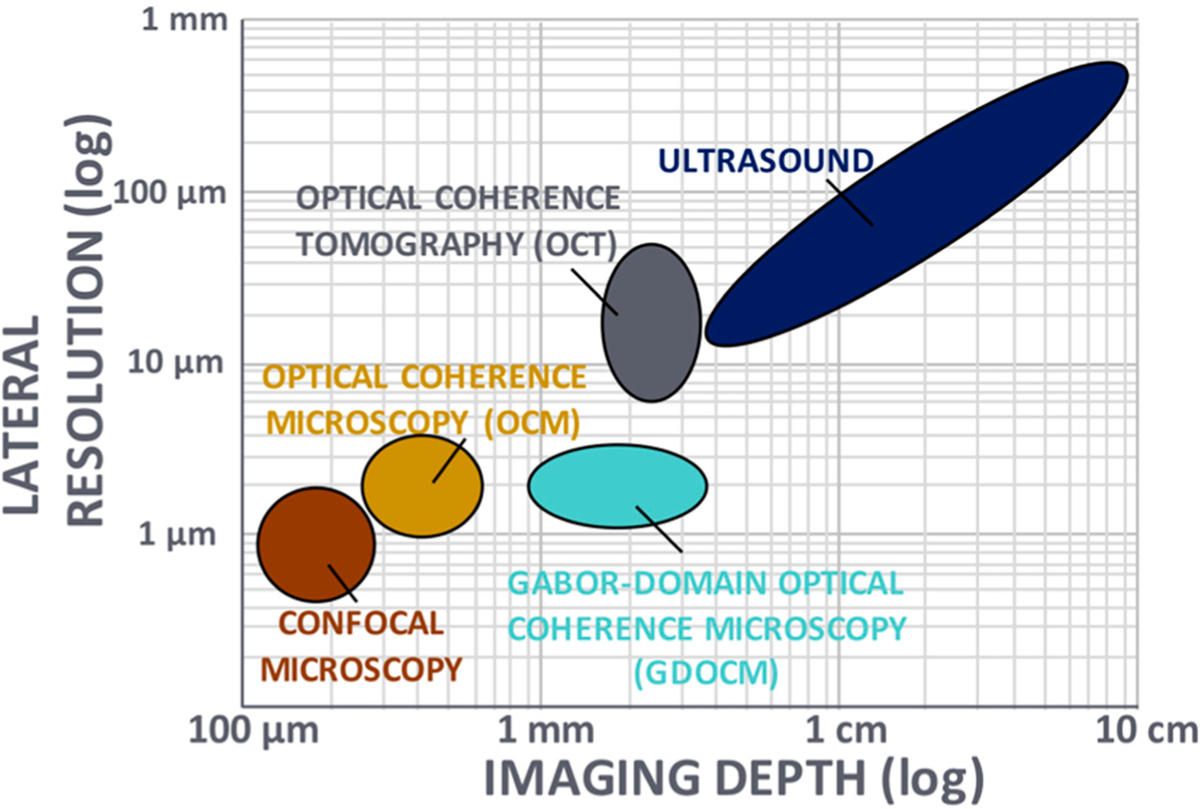
Noninvasive imaging techniques (adapted from [1]). Ultrasound and optical coherence tomography suffer from insufficient lateral resolution for cellular imaging, while confocal microscopy and optical coherence microscopy suffer from limited imaging depth in tissue. Gabor-domain optical coherence microscopy was introduced to overcome the tradeoff between transverse resolution and depth of focus.

Noninvasive imaging techniques, which include ultrasound, optical coherence tomography (OCT), and confocal microscopy, are routinely used in clinical applications for providing insight on tissue structural morphology These methods face a tradeoff between spatial resolution and depth of imaging, as depicted in Figure 1, in which the application space is shown in log-scale for the two key parameters of transverse resolution and imaging depth. These two parameters determine what kind of features can be imaged—including cellular and subcellular—and at what depth inside of the tissue. With typical cellular structures having a size of 10–20 μm, a resolution <5 μm is desired to visualize the cellular morphology A depth of imaging of ~1 mm or more is advantageous to track cellular changes in tissue induced by various diseases.

OCT is an optical imaging technique based on low-coherence interferometry with an axial resolution on the micrometer-scale and lateral resolution limited to tens of micrometers [2], OCT produces cross-sectional views of tissue up to a depth of several millimeters. In OCT, transverse and axial resolutions are decoupled. OCT—in particular spectral domain OCT (SD-OCT)—is widely used in ophthalmic and cardiovascular applications [2,3].

**Figure 2.**
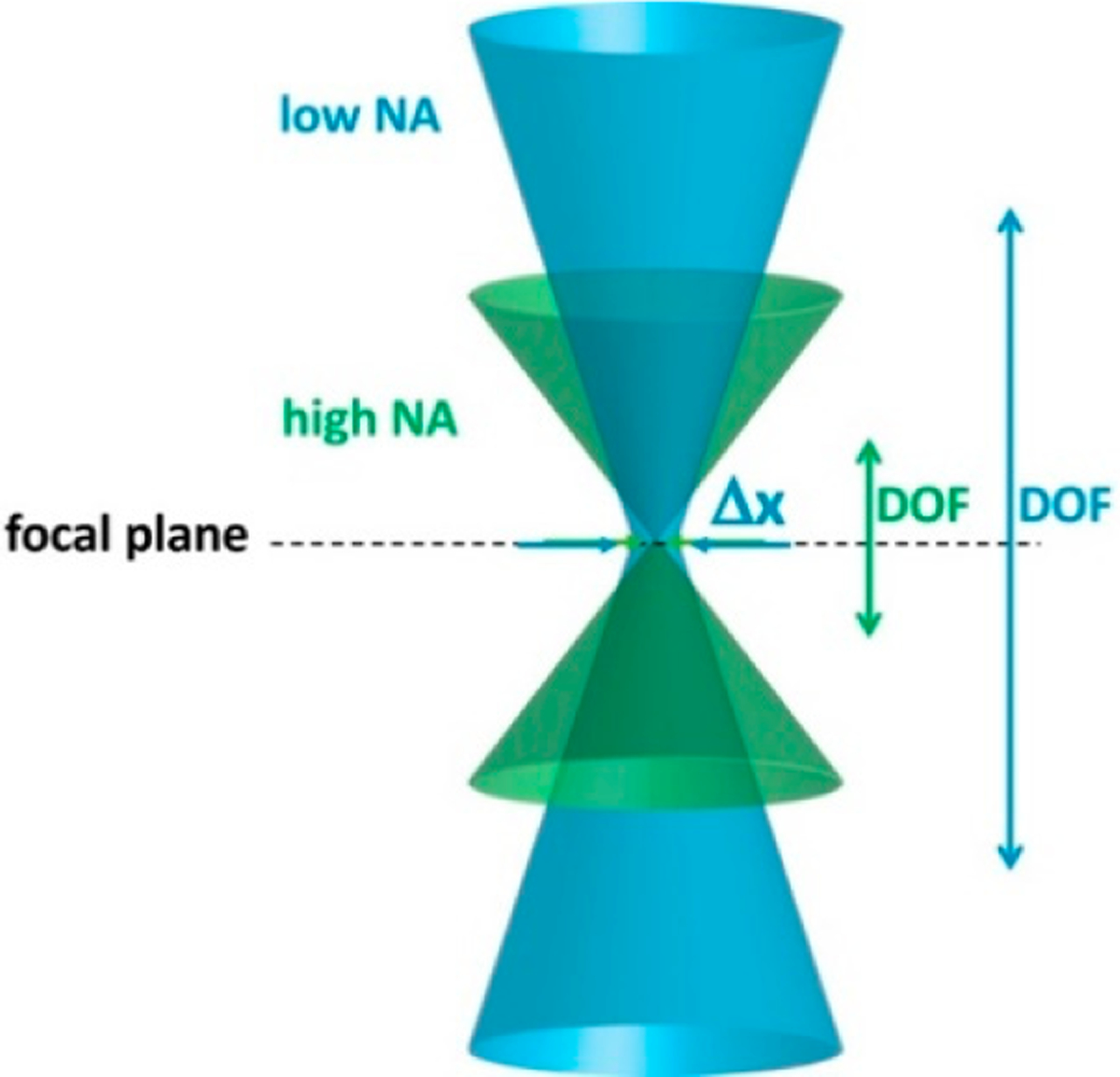
Tradeoff between lateral resolution (Δ*x*) and depth of focus (DOF) in an optical imaging system. NA: Numerical aperture.

Confocal microscopy, with micron or submicron lateral resolution, has depth of imaging limited to tens of micrometers [4]. Optical imaging system, including OCT and confocal microscopy, face a trade-off between lateral resolution and depth of imaging. To increase the lateral resolution Δ*x*, the numerical aperture (NA) is increased, resulting in shallower depth of focus (DOF), as illustrated in Figure 2.

Optical coherence microscopy (OCM) was introduced as a variant of OCT to achieve micrometer-scale resolution [5]. OCM uses a higher NA objective (i.e., ~0.2) than conventional OCT (i.e., ~0.04), and can produce cellular imaging at the expense of a reduction in depth of focus (100–200 μm). OCM variants include point-canning OCM and full-field OCM [6–8]. In full-field OCM, more often referred to as full-field OCT (FF-OCT), en face images are acquired. Computational approaches for extending the depth of focus in OCM have been proposed successfully [9,10].

The choice of the light source in OCT and OCM has direct impact on both transverse and axial resolutions, as well as on imaging depth, since transverse resolution is linearly proportional to the central wavelength, axial resolution is inversely proportional to the bandwidth, and longer wavelengths penetrate deeper into tissue. Superluminescent diodes (SLDs) [11] are broadly used in OCT due to availability in the 800,1300, and 1500 nm spectral bands, relatively large bandwidth (typically 50–100 nm), affordability, and increased brightness (at the expense of bandwidth) over thermal sources [12], An advantage of fluorescence-based sources, which have been employed in FF-OCT in conjunction with pulsed illumination to reduce motion artifacts, is the smoothness of the spectrum, yet their applicability is limited due to cost and requirement of a high-power laser excitation source [13]. Light-emitting diodes (LEDs), have been described to produce submicron axial resolution in FF-OCT with spatial resolution and sensitivity equivalent to those obtained with halogen sources [14]. Supercontinuum sources offer ultrawide bandwidths (more than 1000 nm) and brightness an order of magnitude greater than SLDs, but are still more expensive than SLDs [15]. Yet, they provide superior precision in a thickness estimation task [16,17]. Advances in swept sources have led OCT to achieve multi-MHz acquisition speeds [18], while at the same time they often suffer from jitter that may cause significant uncertainty in a class thickness estimation task [16]. To date, visible light OCT mainly employs spatially coherent light sources, however broadband spatially incoherent light sources have been demonstrated successfully [19].

## 3. Gabor-Domain Optical Coherence Microscopy

**Figure 3.**
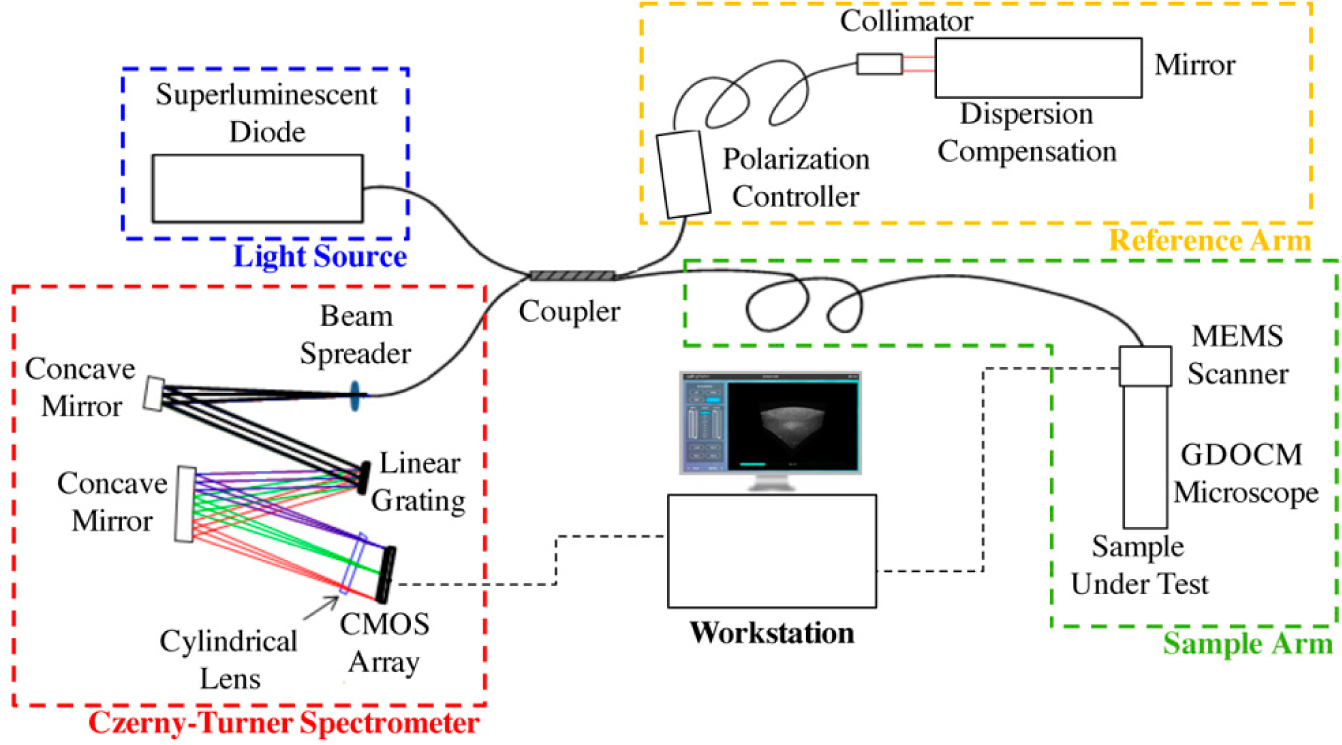
Schematic of a Gabor-domain optical coherence microscopy (GDOCM) microscope consisting of a fiber-based Michelson interferometer. CMOS: Complementary metal oxide semiconductor; MEMS: Microelectromechanical systems.

Gabor-domain optical coherence microscopy (GDOCM) is a high transverse resolution variant of spectral domain optical coherence tomography (SD-OCT) [20]. A schematic of the GDOCM system is shown in Figure 3.

**Figure 4.**
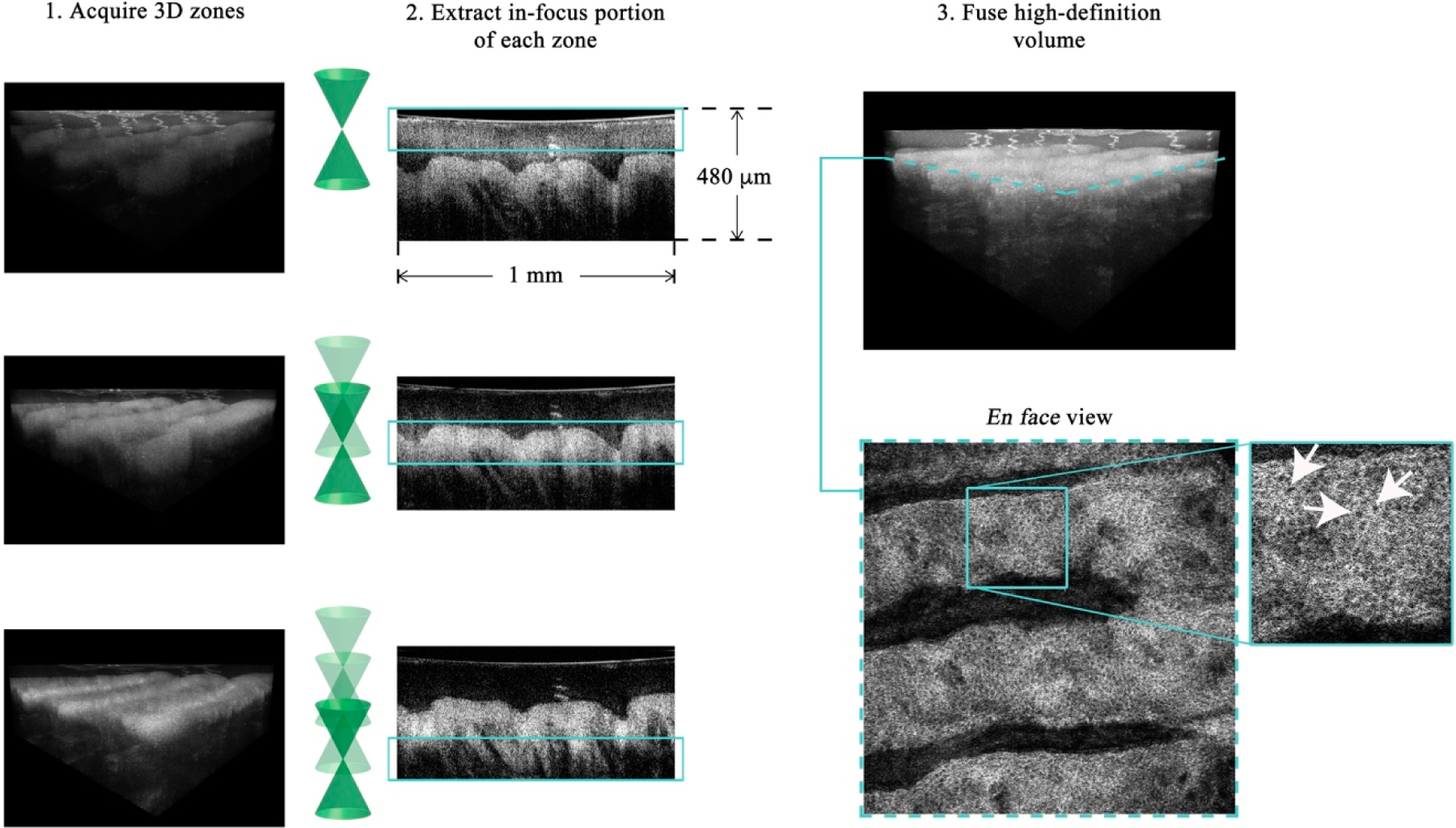
Example of GDOCM image acquisition and fusing of a human fingertip. The field of view is 1 × 1 mm. After acquiring the desired number of zones (three in this case), the in-focus portions are extracted and fused in a high-definition volumetric image, which achieves cellular resolution throughout the volume. An en face view of the dermoepidermal junction (corresponding to the dashed teal line in the 3D view), with basal cells clearly visible (white arrows), is shown.

In GDOCM, the choice of wavelength range in the near infrared was made prioritizing lateral resolution over imaging depth and penetration into tissue. The light source is a superluminescent diode with a center wavelength of 840 nm and 100 nm bandwidth, yielding axial resolution of 3.1 μm in air and 2 μm in tissue (at a refractive index of 1.3). A 50:50 fiber coupler is used for the interferometer. The reference arm performs optical path length matching and dispersion compensation by incorporating a dispersive element [21]. Polarization controllers are used to maximize fringe interference by matching the polarization of the light in the two arms of the interferometer. Optionally, the dispersion and polarization adjustments can be performed to compensate changes in dispersion and polarization introduced by the sample itself. The spectral interference signal is acquired at a line rate of 80 kHz, with imaging depth in the sample greater than 2 mm with a custom Czerny-Turner spectrometer, which includes a reflective dispersive element and a line camera with CMOS (complementary metal oxide semiconductor) sensor. The spectrometer design eliminates coma by incorporating two off-axis spherical mirrors, and a cylindrical lens is used to compensate astigmatism in the beam introduced by the off-axis mirrors [22]. A 2 μm transverse resolution is achieved over a field of view of 2 × 2 mm in the GDOCM microscope design with a numerical aperture of 0.2, with experimental depth of focus of ~100 μm [23]. A compact dual-axis MEMS (microelectromechanical system) mirror integrated in the microscope is used to scan the beam over the 2D field of view [24]. The microscope can be operated in two modalities, to be selected for the desired application: In contact with the sample (with gel medium to create optical contact between the distal surface of the microscope and the sample), or with a working distance of 15 mm. The contact imaging modality may be more advantageous to maximize signal collection in highly scattering tissues such as skin, while the 15 mm working distance is preferred for ophthalmic applications, or for imaging areas that are more difficult to reach with a contact probe, such as certain locations on the face. In order to obtain micron-resolution imaging over the entire volume, multiple volumetric images of the sample (termed zones) are acquired, each corresponding to a different focusing; depth. A good rule of thumb for the number of zones to be acquired is to consider the ratio between total sample depth and the depth of focus; for example, given the depth of focus of ~100 μm, for a total sample depth of 600 μm, six zones are required. A liquid lens integrated in the optical design of thief microscope achieves dynamic refocusing with no moving parts over a 2 mm range [25]. The in-focus portions of each volumetric zone are extracted and merged together to produce a high-definition volume with invariant 2 μm resolution, both axially and transversally. The process of combining together the in-focus portions of the zones is referred to as fusing. Fusing can be performed either in the spatial or spectral domains [26,27]. as example of a GDOCM image of a human fingertip acquired with three zones and the corresponding fusing procedure is shown in Figure 4.

Parallel processing of the acquired data on graphics processing units (GPUs) achieves near real-time visualization of the volumetric images [28].

**Figure 5.**
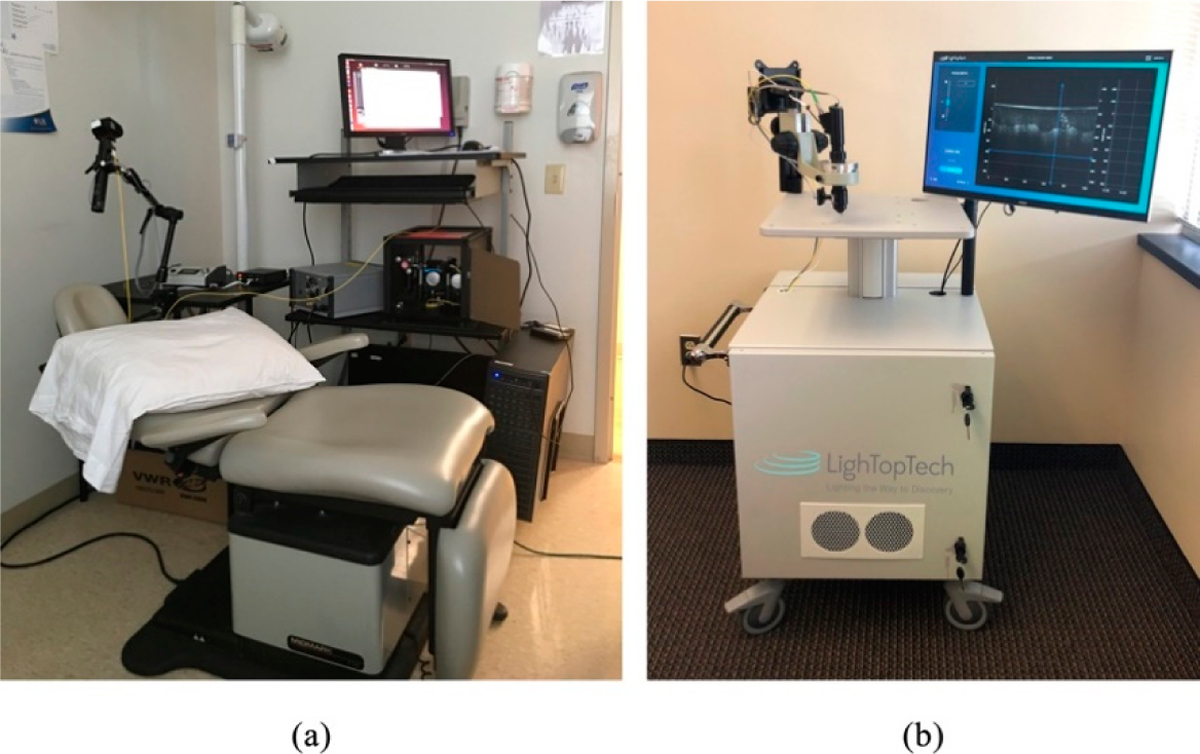
(**a**) GDOCM system in use at the University of Rochester Department of Dermatology in its first in vivo study in the fall of 2015; (**b**) 2018 version of GDOCM (LighTopTech Corp. GDOCM 4D™).

The 2015 and 2018 implementations of GDOCM are shown in Figure 5. A standalone cart houses the entire GDOCM system. While the first prototype shown in Figure 5a included several elements mounted on a breadboard assembly, the instrument shown in Figure 5b was entirely re-engineered with precision mechanics for robust and reliable operation, and incorporates a custom software for semiautomated image acquisition, and 3D image visualization and analysis.

**Figure 6.**
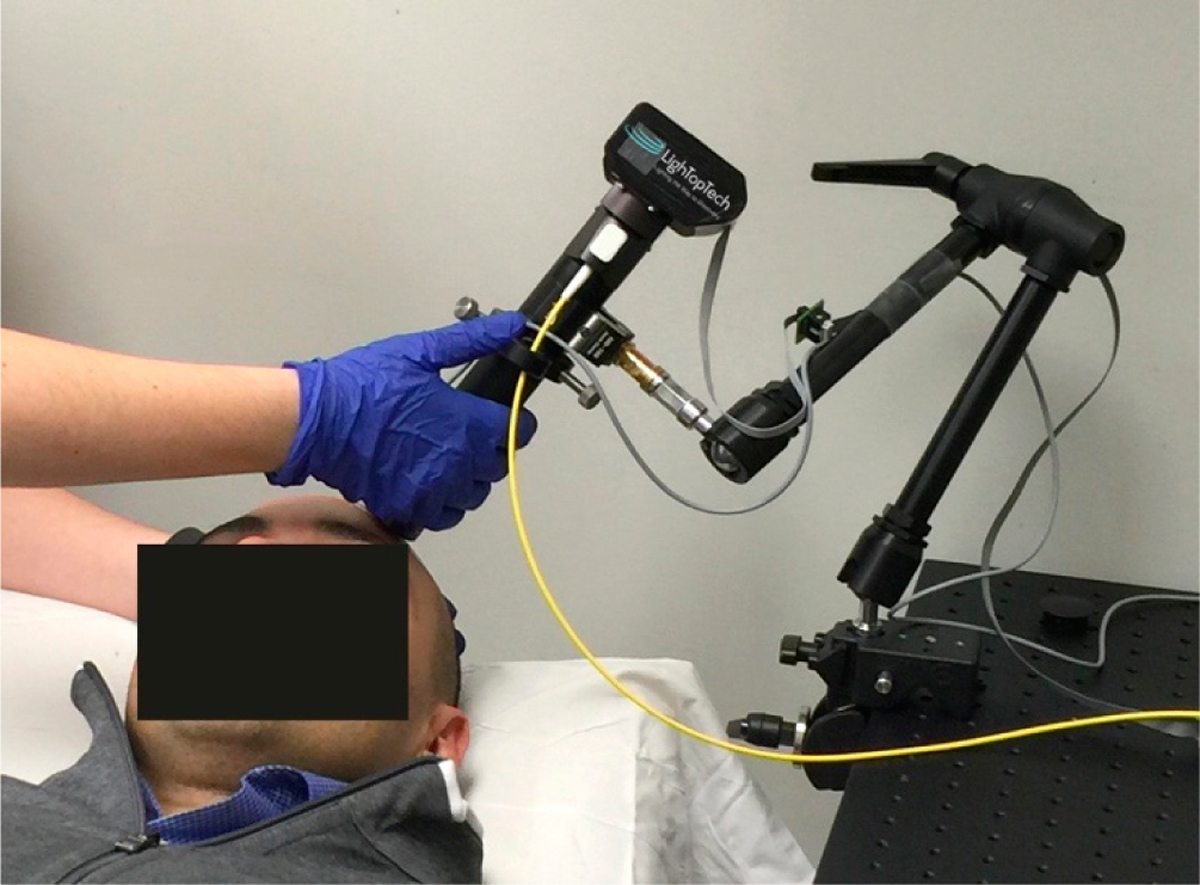
Handheld GDOCM microscope. A mechanical arm can be used to reduce operator hand tremor.

The microscope can be operated either as a handheld or with an articulated arm (see Figure 6, for an example of contact mode with a mechanical arm). The use of a mechanical arm is desired to minimize motion artifacts introduced by operator hand tremor.

A comparison of the imaging performance of confocal microscopy, spectral domain optical coherence tomography, optical coherence microscopy, full-field optical coherence tomography, and Gabor-domain optical coherence microscopy is reported in Table 1. The performance of the various imaging modalities relates to the chart in Figure 1, with SD-OCT suffering from limited transverse resolution while offering excellent imaging depth of several millimeters, while confocal microscopy, OCM, and FF-OCT achieve subcellular resolution over a limited imaging depth. GDOCM balances these parameters to achieve cellular resolution over an imaging depth of 2 mm. SD-OCT, OCM, and GDOCM, being spectral domain OCT systems, have a cross-sectional image orientation, while CM and FF-OCT (time domain OCT) have an en face image orientation. A main limitation of confocal microscopy, unlike OCT, is that the sectioning in depth is set by the NA of the objective lens. Contact operation is typically required with clinical confocal microscopes, however contactless implementations have been proposed. Higher sectioning is obtained with higher NA, yet this occurs at the expense of DOF. Because axial sectioning in OCT is independent of the NA, since it is set by the spectral bandwidth, both higher axial sectioning and larger DOF are possible with OCT. Furthermore, it may be difficult to assess the depth of imaging with confocal, as only en face planes are acquired. SD-OCT acquires depth scans, from which the volume is assembled, and the depth of any en face plane is directly acquired from the depth scan. While the methods that offer higher transverse resolution typically have limited FOV, mosaicking is often used to provide wide FOV imaging, naturally at the expense of acquisition time.

## 4. Applications

Originally developed for ophthalmic applications to image the posterior segment of the eye [3,33–35] and further enhanced with OCT angiography [36,37], OCT has found successful applications in the anterior segment of the eye [30], as well as in a number of fields, including dermatology [38,39], oncology [40–46] and dentistry [47]; in endoscopic form it has been applied to cardiology [48,49], gastroenterology [50,51], and pulmonology [52–54]. Numerous embodiments of functional OCT [55], including Doppler OCT and polarization-sensitive OCT [56,57], as well as optical coherence elastography [58–64], multimodal fluorescence-OCT [2,65–67] and spectroscopic OCT [68], have been developed to enhance the structural information obtained with OCT with properties related to tissue function.

**Figure 7.**
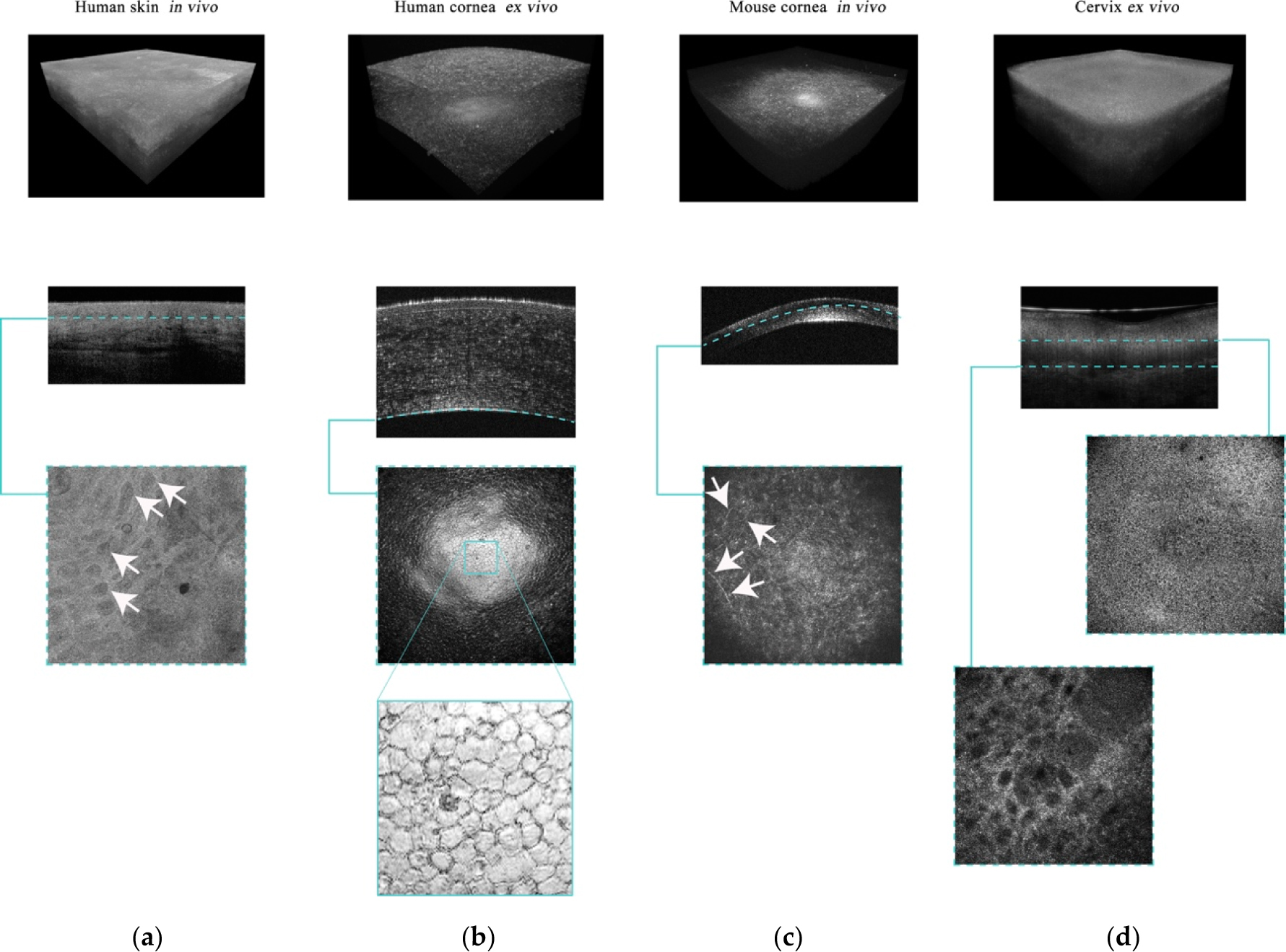
Representative 3D, cross-sectional, and en face views for different GDOCM applications: (**a**) In vivo human skin with basal cell carcinoma (BCC), (**b**) ex vivo human cornea, (**c**) in vivo mouse cornea, and (**d**) ex vivo human cervical tissue. The images have a field of view of 1 × 1 mm. The arrows in the en face view of in vivo human skin indicate the BCC. The en face view of the human cornea shows the endothelium, a single layer of cells lining the posterior surface of the cornea, with the endothelial cells clearly visible. The arrows in the en face view of a mouse cornea acquired in vivo indicate the corneal nerves. In the images of uterine cervix, cervical stroma, basement membrane, and cervical epithelium are visible

To date, GDOCM has been used in a number of medical applications, including human skin ex vivo [69,70] and in vivo [71], human corneas ex vivo [72], mouse cornea in vivo, and cervical tissue ex vivo [73], as well as industrial applications [74,75]. When reviewing the images, having access to the 3D views offers additional insights into tissue morphology; the en face views are useful to visualize cellular structures. Representative images of human skin, cornea, and cervical tissue are shown in Figure 7. The depth cross-sections can be directly related to traditional histology slices, since they are presented in the same orientation. The en face views, which are in the traditional orientation of microscopy, including confocal microscopy, highlight the presence of cellular structures, including endothelial cells (Figure 7b, bottom) and corneal nerves (Figure 7c, bottom). Various diseases that cause disruption of the cellular network, such as basal cell carcinoma (BCC) and cervical dysplasia, can be assessed, such as in Figure 7a,d. Various measurements can be conducted on the 3D GDOCM images to extract relevant parameters, such as quantifying the thicknesses of various sublayers of the tissue, and estimating the cell density [76].

## 5. Conclusions

A number of developments are under course to further enhance the cellular-resolution imaging capabilities of GDOCM. These include applying machine learning to automatically segment features of interest of the image; adding functional capabilities to enhance GDOCM′s structural imaging at the microscopic level, such as fluorescence and elastography; and thickness estimation of nano-scale layers.

## Figures and Tables

**Table 1.** Comparison of noninvasive imaging technologies. CM: Confocal microscopy; FF-OCT: Full-field optical coherence tomography; SD-OCT: Spectral domain optical coherence tomography; OCM: Optical coherence microscopy; GDOCM: Gabor-domain optical coherence microscopy.

Technology	Axial Resolution (μm)	Transverse Resolution (μm)	Imaging Depth (mm)	Field of View (mm)	Image Orientation	Contact
**CM** [29]	7.6	1–2	<0.1	0.4 × 0.4	En face	Yes
**SD-OCT** [30]	1–10	10–20	6	6–16	Cross-sectional	No
**OCM** [31]	1.5	1.5	<0.2	0.8 × 0.8	Cross-sectional	Not required
**FF-OCT** [14,32]	0.7–7.7	1.7–2	<1	0.9–1.3 × 0.9–1.3	En face	Not required
**GDOCM**	2	2.6	2.5	1.5 × 1.5	Cross-sectional	Not required
